# Exploring intrinsic MR signal relaxation in acute RF ablation lesions using T2 mapping and IR-SSFP CINE imaging

**DOI:** 10.1186/1532-429X-15-S1-O87

**Published:** 2013-01-30

**Authors:** Venkat Ramanan, Haydar Celik, Sudip Ghate, Jennifer Barry, Nilesh R Ghugre, Vivian Leber, Jeff A Stainsby, Andriy Shmatukha, Mohammed Shurrab, Eugene Crystal, Graham Wright

**Affiliations:** 1 Imaging Research, Sunnybrook Research Institute, Toronto, ON, Canada; 2 Arrhythmia Services, Sunnybrook Health Sciences Centre, Toronto, ON, Canada; 3 GE Healthcare, Toronto, ON, Canada

## Background

Cardiac MR has been used successfully in RF ablation therapies for arrhythmias, both for procedural planning and for post-ablation lesion imaging. Non-enhanced imaging, though it has a lower SNR, has advantages over Gd-enhanced techniques mainly because contrast kinetics and dosage issues are avoided. Previously T2-weighted imaging was found to be more sensitive than T1-weighted imaging [1]. In this study, we performed non-enhanced T2 mapping and an inversion-prepared SSFP CINE imaging to characterize intrinsic relaxation behavior in acute lesions.

## Methods

Using approved animal protocols, 15 ablation lesions were created using irrigated catheters, on the endocardium of the left ventricle in 8 healthy pig models under X-ray and Carto-XP guidance. After transferring the animal to the MR suite, we performed T2 mapping using a multi-echo Fast-Spin-Echo (MEFSE) sequence [2] and T1* characterization using a 2RR-IR-SSFP CINE method [3]. Both MEFSE and IRSSFP were done within 30-60 mins of creating the lesions. Then Gd-DTPA (Magnevist, 0.2 mmol/kg) was injected and late-Gd-enhancement (LGE) scans were done multiple times from 10-60 min after contrast injection. The animals were then sacrificed and the hearts were preserved for pathology.

MEFSE images were fitted using a standard 2-parameter fit for M0 and T2. The M0 term here is a function of both true M0 and T1, because it was a single-RR (RR = 700 ms) sequence. IRSSFP signal recovery is determined by T1* (dependent on both T1 and T2). Ablation extent was confirmed by LGE and pathology. The T2 and M0 maps and IRSSFP images were then analyzed using an ROI based analysis to compare the signal in 3 zones (ablation lesion, adjacent edema and remote tissue).

## Results

The T2 and relative M0 values are given in Table 1. On T2-maps there was a broad area of increased T2 in and around the ablation, likely due to edema; this area was much larger than lesion extent on pathology [4]. However the M0-maps seem to correspond closely with the actual border of ablation. Contrast in T1*-weighted IRSSFP images also clearly depicted the actual border of the ablation seen on pathology [4]. T1* is about 315ms in the remote zone while in ablation zone it was significantly lower at 270ms.

**Table 1:** T2 and M0 in remote, edema and ablation zones.

Area of myocardium	T2 in ms: Mean (Range)	M0 map: Percentage increase of mean signal of ROI compared to remote zone. [ 100*(ROI- ROI_remote)/ROI_remote ]
Remote zone	47 (42-53)	0%
Edema zone	79 (65-103)	31%
Ablation zone	83 (69-98)	85%

The table shows the values of T2 and M0 in the ablation, edema and remote zones. The ablation zone was defined as the area of high signal intensity on M0map near the ablation as confirmed by LGE and pathology. The edema zone was defined as the area of high T2 values near the ablation. It surrounds the ablation zone. The remote zone was defined on the same slice far from the ablation. Note that in the ablation zone, M0 increased by 85%, in the edema zone about 30%. The T2 values increased in the ablation and edema zones by about the same amount (30-35 ms).

## Conclusions

The M0 and T2 maps appear to depict actual lesion area and edema respectively. T1*-weighted IRSSFP generally gives more robust visualization of the ablation lesion within a breath-hold. The high signal of the lesion core in T1 and T1*-weighted images might be due to methemoglobin, which is created upon heating blood [5]. In summary, T1-weighting seems to delineate the acute lesion core while T2-weighting seems to depict the overlying edema.

## Funding

We gratefully acknowledge support from GE Healthcare, the Ontario Research Fund, and Canadian Institutes of Health Research.

**Figure 1:**
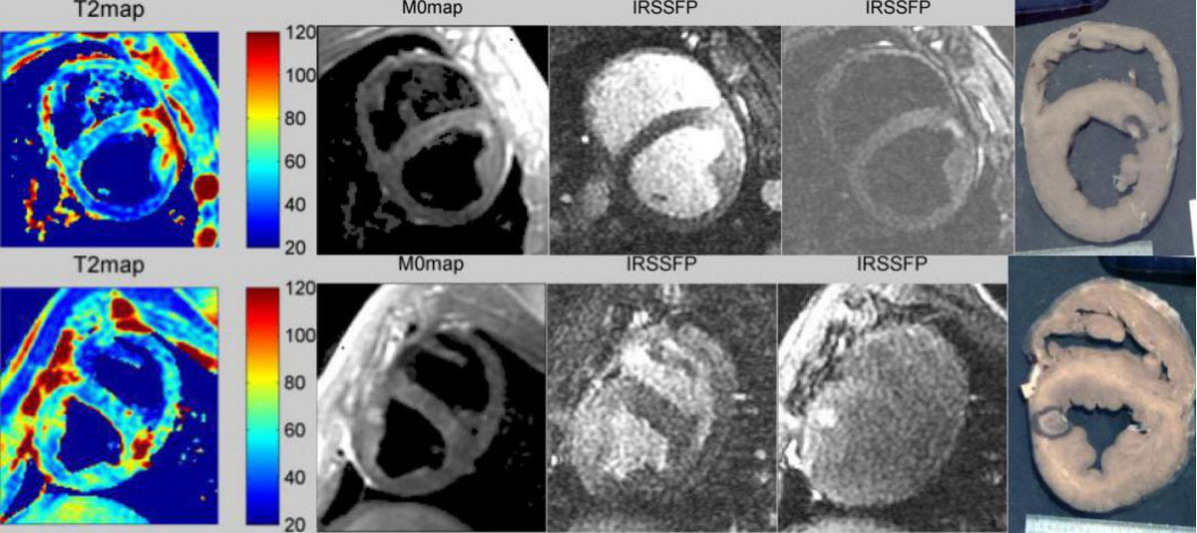
The two rows show ablations in two pigs. The first two columns show T2maps and M0maps fitted from T2-mapping MEFSE sequence. The next two show IR-SSFP CINE images for two phases with different inversion times and the last one shows gross pathology of the lesion. The T2maps show a large area of diffuse T2 enhancement in and around the ablation region possibly due to edema. The M0 maps closely correspond in size and location to the ablation seen in gross pathology. Note that the M0map is T1-weighted. IRSSFP shows two different heart-phases from CINE reconstruction. The first phase is acquired in the first-RR with a short inversion time (TI=116 ms) and therefore has bright blood while the second phase is acquired later in the second-RR (TI=616ms) when the blood signal is nulled. Signal enhancement around the ablation region in the first phase corresponds to edema and in the second phase to the actual border.
